# Clinical features in a large Iranian family with a limb-girdle congenital myasthenic syndrome due to a mutation in *DPAGT1*

**DOI:** 10.1016/j.nmd.2013.03.003

**Published:** 2013-06

**Authors:** Keivan Basiri, Katsiaryna Belaya, Wei Wei Liu, Susan Maxwell, Maryam Sedghi, David Beeson

**Affiliations:** a Neurology Department, Neuroscience Research Center, Isfahan University of Medical Sciences, Isfahan, Iran; b Medical Genetics Laboratory, Alzahra University Hospital, Isfahan University of Medical Sciences, Isfahan, Iran; c Neurosciences Group, Weatherall Institute of Molecular Medicine, The John Radcliffe, Oxford, UK

**Keywords:** Congenital myasthenic syndrome, *DPAGT1*, Mutation, Clinical features

## Abstract

Mutations in *DPAGT1* are a newly recognised cause of congenital myasthenic syndrome. *DPAGT1* encodes an early component of the N-linked glycosylation pathway. Initially mutations in *DPAGT1* have been associated with the onset of the severe multisystem disorder – congenital disorder of glycosylation type 1J. However, recently it was established that certain mutations in this gene can cause symptoms restricted to muscle weakness resulting from defective neuromuscular transmission. We report four cases from a large Iranian pedigree with prominent limb-girdle weakness and minimal craniobulbar symptoms who harbour a novel mutation in *DPAGT1*, c.652C>T, p.Arg218Trp. This myasthenic syndrome may mimic myopathic disorders and is likely under-diagnosed.

## 1 Introduction

The congenital myasthenic syndromes (CMS) constitute a group of rare genetic disorders that result in impaired synaptic transmission at the neuromuscular junction. The number of genes shown to harbour mutations that underlie CMS continues to grow with at least 16 described [1,2]. Whereas many of the CMS involve mutations in proteins with well-defined functions at the neuromuscular junction, the two most recently identified causes of CMS are mutations in *GFPT1* [3] and *DPAGT1* [4] both of which encode ubiquitously expressed proteins. *DPAGT1* encodes dolichyl-phosphate (UDP-*N*-acetylglucosamine) *N*-acetylglucosaminephosphotransferase 1, an enzyme involved in the early steps of the asparagine-linked (N-linked) glycosylation pathway [5], and it is proposed that impaired glycosylation of the muscle acetylcholine receptor is a primary pathogenic mechanism in this form of CMS [4]. A number of beneficial treatments are available for CMS, but appropriate treatment often depends upon recognition of a myasthenic disorder, knowledge of which gene is mutated, and an understanding of the underlying molecular mechanism [6]. Many of the recently identified forms of CMS have a predominantly proximal pattern of muscle weakness with onset in childhood that can lead to a diagnosis of non-specific myopathy, congenital myopathy, or congenital muscular dystrophy prior to a definitive genetic diagnosis [2]. Here we provide case reports for a large Iranian family harbouring a previously unreported mutation in *DPAGT1*, which both expands the described phenotype and may help recognition of this disorder.

## 2 Case report

### 2.1 Mutation analysis

**Fig. 1:**
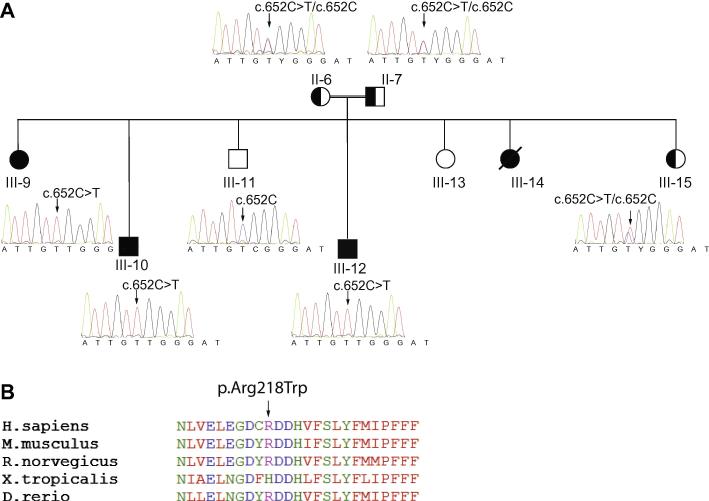
A. Sanger sequencing of exon 5 of *DPAGT1* showing co-segregation of c.652C>T, (p.Arg218Trp), with disease in the four affected individuals within the pedigree. B. Conservation of the amino acid sequence of *DPAGT1* between species in the region harbouring the mutations p.Arg218Trp.

No abnormal variants were detected in *DOK7* or *GFPT1* which give rise to CMS with a predominantly limb-girdle pattern of muscle weakness [7,3]. However, subsequent bi-directional sanger sequence analysis of PCR-amplified exonic regions and their flanking sequences in DNA from the proband (Case 2) detected a previously unreported homozygous variant mutation in exon5 of *DPAGT1* (RefSeq: NM_001382.3) [5]. The mutation, c.652C>T, results in the missense amino acid substitution p.Arg218Trp. This newly identified mutation was found to segregate with affected members within this large consanguineous family and is consistent with recessive inheritance of CMS due to *DPAGT1* mutations [4] (Fig. 1A). The mutation is of a residue conserved in mammals and zebrafish (see Fig. 1B), and is predicted to be damaging using the ‘Mutation Taster’ program (http://www.mutationtaster.org/) [7]. The *DPAGT1* c.652C>T variant is not present in 1000 Genomes Project (http://browser.1000genomes.org) [8], the Exome variant server (http://evs.gs.washington.edu/EVS/) [9], or the dbSNP database (http://www.ncbi.nlm.nih.gov/projects/SNP/) [10] consistent with designation as a pathogenic mutation.

*Case 1* (*III-14*) was a female from this large Iranian family who was symptom-free up to school age, when she presented with mild and fluctuating proximal weakness of lower extremities. Diagnosis of myasthenia gravis (MG) was proposed when she was 7 years old and administration of pyridostigmine, prednisone and physical and occupational therapy resulted in partial relief. She was able to perform almost normally in daily activities for the next five years. Aged 15 she was hospitalised with respiratory failure requiring ITU. Increase in pyridostigmine and prednisolone led to partial recovery with no history of hospitalisation for the subsequent few years. The patient again required hospital intervention aged 24, when her symptoms worsened following a viral illness. At this time azathioprine was added to her treatment regimen.

On examination, aged 25, she had severe weakness of her proximal muscles. Deep tendon reflexes were reduced throughout. Antibodies to the acetylcholine receptors were not present. Electrophysiological testing revealed abnormal decrement (up to 32%) on repetitive nerve stimulation in keeping with a myasthenic disorder. Myopathic potentials were seen on standard needle EMG examination of all muscles tested and were most prominent proximally. Fibrillations and positive sharp waves were also detected on EMG. Nerve conduction studies were normal. CT of the thymus was normal. A muscle biopsy from the right vastus lateralis showed a reduced number of muscle fibres and replacement with fat and connective tissue. The remaining muscle fibre size was variable and some demonstrated centralisation of nuclei. No tubular aggregates were detected.

The withdrawal of prednisone and azathioprine and symptomatic treatment with pyridostigmine resulted in the patient regaining much of her normal strength. However, the following year the patient died during a further respiratory crisis.

*Case 2* (*III-9*), a sister of case 1, at age 17 had sudden onset of muscle cramps, severe fatigue and fluctuating ptosis. She was treated for myasthenia gravis and despite thymectomy, no significant improvement was seen. On examination aged 28 she had mild proximal weakness of her lower limbs. Deep tendon reflexes were normal. There was significant decrement in limb (40%) and facial muscles (26%) on repetitive nerve stimulation. Needle EMG examination showed a myopathic pattern with no evidence of active denervation. A muscle biopsy of Vastus lateralis muscle disclosed variation in fibre size, centralisation of nuclei and replacement of fibres with fatty tissue, without tubular aggregates. Serum creatine kinase was normal. Tests for acetylcholine receptor antibodies were negative.

*Case 3* (*III-10*), a brother, first presented with frequent falls at the age of 3 years, and had a history of mild weakness of extremities and muscle wasting. Muscle cramps started aged 24. On examination aged 26 he had mild weakness in proximal limbs, with sparing of ocular muscles. Ankle reflexes were undetectable. Tests for acetylcholine receptor antibodies were negative but pyridostigmine resulted in symptomatic relief. On repetitive nerve stimulation significant decrement was detected in the small muscles of the hands (29–33% in ADM and APB), but not in facial muscles. A myopathic pattern was seen on needle EMG examination of proximal limb muscles.

*Case 4* (*III-12*), a brother of the previous cases, first noticed weakness with difficulty climbing stairs aged 13 years, and was also noted at this time to have an abnormal gait. On examination aged 34 he had mild dysarthria, mild scoliosis, and lumbar hyperlordosis. Ankle reflexes were reduced. There was mild weakness of neck flexor, arm abduction and extension and mild weakness of hip extensors and abductors. There was also mild fatiguable ptosis.

## 3 Discussion

We present a large consanguineous Iranian family in which a limb-girdle myasthenia co-segregates with mutations in the newly identified CMS-associated gene, *DPAGT1*. The affected family members expand the phenotypic features associated with mutations in *DPAGT1* and highlight clinical characteristics that differ from the more common forms of CMS.

Clinical features associated with myasthenic syndrome due to mutations in *DPAGT1* [4] include fatiguable weakness that predominantly affects proximal muscle groups, spares ocular and facial muscles, and is responsive to anticholinesterase medication. Onset is in childhood usually delayed beyond infancy and affected individuals may have myopathic features associated with tubular aggregates on muscle biopsy [4]. The cases described here demonstrate a similar overall phenotype. They show a more variable age of onset (between 3 and 17 years) than previously reported and a marked contrast in disease severity within a consanguineous family. None had mild learning difficulties that can occur in this disorder. Case 1 suffered re-occurring life-threatening respiratory crises which we have not previously seen in individuals with *DPAGT1* mutations. Marked fluctuations in weakness are noted and muscle cramps, which are a common feature of *DOK7* CMS [11,12] were prominent symptom for Cases 2 and 3. Repetitive nerve stimulation demonstrates marked decrement and thus indicates impaired neuromuscular transmission. However EMG revealed myopathic findings, and in combination with the changes seen on the muscle biopsies points to a myopathic component in the disorder. Of note for Case 3, decrement was evident for the small muscles of the hand which can show marked weakness in *DPAGT1* CMS, but was not seen in facial muscles that tend to be spared. Surprisingly, tubular aggregates were not identified in muscle biopsies from these cases, indicating that their presence cannot be used as a definitive marker for *DPAGT1* CMS. A comparable situation is seen in limb-girdle CMS due to *GFPT1* mutations where tubular aggregates are a common feature but they are not always present [13].

As well as causing CMS, certain mutations in *DPAGT1* have been reported to cause congenital disorder of glycosylation type 1J (CDG type 1J) [14–17]. This is one of a group of disorders caused by defects in the formation or processing of glycoproteins or glycolipids [18]. Rare cases of CDG type 1J due to *DPAGT1* mutations have been reported as a severe multisystem disorder with symptoms including intractable seizures, congenital cataracts, mental retardation, and developmental delay with microcephaly [14,15], or with severe foetal hypokinesia with death in infancy or early childhood [16,17].

The full phenotypic spectrum for mutations in *DPAGT1* is likely to emerge with the advent of next generation sequencing techniques, and as yet it is not clear why symptoms in the patients we describe are largely restricted to muscle and in particular to the neuromuscular junction. Mutations in *DPAGT1* are thought to impair AChR subunit glycosylation leading to reduced assembly and transport of the AChR into the postsynaptic membrane, and thus reduced endplate AChR number [4], which is consistent with a beneficial response to pyridostigmine. The relatively late onset of *DPAGT1* CMS, fluctuating muscle weakness, response to anticholinesterase medication and decremental response on repetitive nerve stimulation may suggest seronegative myasthenia gravis. Conversely, onset in childhood and absence of ocular or facial weakness, and features on muscle biopsy may suggest a non-specific myopathy without a myasthenic component. It is likely there are further patients with *DPAGT1* CMS who currently carry a diagnosis of seronegative myasthenia gravis or an undefined myopathic disorder. Recognition of these cases should facilitate a genetic diagnosis and an appropriate therapeutic strategy.
